# Long-term effectiveness and safety of infliximab and golimumab in ankylosing spondylitis patients from a Canadian prospective observational registry

**DOI:** 10.1186/s41927-020-00158-z

**Published:** 2020-11-15

**Authors:** Proton Rahman, Michael Starr, Derek Haaland, Louis Bessette, Michelle Teo, Emmanouil Rampakakis, Allen J. Lehman, Francois Nantel

**Affiliations:** 1 Memorial University, St. John’s, NL Canada; 2 McGill University, Montreal, QC Canada; 3 Barrie, ON Canada; 4 Universite Laval, Quebec, QC Canada; 5 Penticton, BC Canada; 6 JSS Medical Research, Montreal, QC Canada; 7 Janssen Inc., 19 Green Belt Dr., Toronto, ON M3C 1N9 Canada

**Keywords:** Ankylosing spondylitis, Axial spondyloarthritis, Registry, Infliximab, Golimumab, Effectiveness, Safety

## Abstract

**Background:**

The objectives of this study were to describe the profile of ankylosing spondylitis (AS) patients treated with either infliximab (IFX) or subcutaneous golimumab (GLM) treatment in Canadian routine care setting along with assessing long-term effectiveness and safety.

**Methods:**

AS patients who were eligible for treatment with IFX or subcutaneous GLM as per their respective Canadian product monographs were enrolled into the BioTRAC registry from 2005 to 2017. The study visits occurred at baseline and every 6 months thereafter. Effectiveness was assessed by changes in clinical outcomes and acute phase reactants. Safety was evaluated by assessing the incidence of adverse events (AEs) and drug survival rates.

**Results:**

A total of 389 IFX- and 421 GLM-treated patients were enrolled. A significant decrease in disease duration at baseline was observed in the IFX cohort, from a median of 8.0 in 2005–2008 to 1.0 years in 2009–2015 (*p* < 0.001). A reduction in baseline BASFI score (*p* = 0.011) and proportion of patients in ASDAS very high disease activity (*p* = 0.004) was also observed over time. Meanwhile, in the GLM cohort, most disease parameters remained similar from 2010 to 2017.

Treatment with both agents significantly improved all disease parameters over time with similar efficacy between the two agents. The incidence of AEs and SAEs were 136 and 131 events/100 PYs and 10.5 and 8.45 events/100 PYs for IFX- and GLM-treated patients, respectively.

**Conclusion:**

Both IFX and GLM treatment in AS significantly reduced disease activity in most outcome measures in a similar fashion and were well tolerated in Canadian routine care.

**Trial registration:**

NCT00741793.

## Background

Ankylosing spondylitis (AS) is a common form of spondyloarthritis (SpA) [1]. It is further classified under the subgroup axial spondyloarthritis (AxSpA), due to the predominant involvement of the spine and/or sacroiliac joints whether it be radiographic or non-radiographic AxSpA. Along with inflammatory back pain, SpA is also characterized by inflammation of tendon/ligament sites of insertion into bone (enthesitis), development of peripheral arthritis in about a third of patients [1, 2] and eventually progression to the fusion of the axial skeleton (ankylosis) in selected patients [3].

Extra-articular manifestations such as ophthalmologic, dermatological and gastrointestinal involvement are also common in AS patients; the prevalence of uveitis, psoriasis and inflammatory bowel disease being 26, 9 and 7%, respectively [4]. Uveitis is the most common extra-articular manifestation, and generally involves the anterior chamber and is unilateral [5].

AS is more common in men than in women [6], and affects a young population starting in adolescence or early adulthood. The overall disease prevalence is estimated to be between 0.1 and 1.4% [7] and varies with the prevalence of the HLA-B27 gene in a given population [8]. In Canada’s largest province (Ontario), the prevalence of AS in 2010 was estimated to be 213/100,000 [9].

Substantial health and economic burdens are associated with AS. Studies investigating quality of life have demonstrated that AS patients report substandard health conditions, especially when considering physical function/mobility and bodily pain [10, 11]. Additionally, significant costs, including out of pocket expenses and missed work, are associated with increasing disease activity and loss of function [12–14]. There is presently no cure for AS, therefore the goal of treatment is to improve the health-related quality of life by reducing pain, improving physical function, and delaying structural damage [15].

Current treatment recommendations include: nonpharmacological intervention (exercise, physical therapy, and lifestyle changes), non-steroidal anti-inflammatory drugs (NSAID; first-line pharmacological treatment) and biologic agents (anti-TNF and anti-IL-17 agents) [16]. While traditional therapy was intended to alleviate pain symptoms, the emergence of biologic agents results in the ability to slow disease progression [17–19]. TNFi demonstrated efficacy in AS, specifically concerning disease activity and functionality, where significant improvements in Bath AS Disease Activity and Functionality Index (BASDAI and BASFI) scores have been observed [20–23]. MRI studies have also shown that anti-TNF treatment can reduce acute inflammation in the spine and sacroiliac joints [24, 25].

The safety profile and effectiveness of anti-TNFs in routine clinical care in AS is not well-established, especially with GLM, and may vary considerably regionally due to differences in patient characteristics and disease management. Therefore, post-approval studies of anti-TNFs are useful for providing real world insights, including any rare and serious adverse events and should be conducted at least at a national level. The current study uses data retrieved from the Biologic Treatment Registry Across Canada (BioTRAC) to describe the profile of Canadian AS patients treated with infliximab (IFX) or golimumab (GLM) in routine clinical practice and to assess the effectiveness and safety of these agents in a real-world setting. An interim analysis of IFX-treated AS patient has been previously reported [26].

## Methods

### Study design

The Biologic Treatment Registry Across Canada (BioTRAC; NCT00741793) was a prospective, multi-centre (140 sites), registry that collected real-world clinical, laboratory, safety, and patient-reported data between 2002 and 2018 among AS, psoriatic arthritis, and rheumatoid arthritis patients treated with IFX, GLM or ustekinumab during routine institutional and private care in Canada. Prior to enrolment, patients were required to provide a written informed consent to participate. Ethics approval was obtained from a central Research Ethics Board (IRB Service, Ontario, Canada) for private practices, and from respective Research Ethics Boards for institutional sites. The study was conducted in accordance with the Declaration of Helsinki. The historical development of the registry and an interim analysis of IFX-treated patients have been described elsewhere [26, 27]. Data from this registry were recently presented at the Canadian Rheumatology Association [28], PANLAR [29] and EULAR [30] 2019 conferences.

### Patient population

For the purposes of this analysis, patients with AS who were eligible for treatment with IFX or subcutaneous GLM as per the Canadian Product Monograph were included. The diagnosis of AS was made by the treating rheumatologists as per standard of care practices. Adult AS patients, either bio-naive (2005–2006) or with ≤1 prior biologic agent exposure (2006–2018), initiating anti-TNF therapy as per their treating physician and the Canadian product monograph, were enrolled and followed for up to 12 years with a study visit at baseline and every 6 months thereafter (a 2-month visit was included from 2005 to 2006 but is excluded from this report).

Patients treated with IFX were enrolled from 2005 until 2015 and followed until 2017 or until treatment termination. GLM-treated patients were enrolled from 2010 to 2017 and they were followed until 2018 or until treatment termination. Following the regulatory approval of GLM for the treatment of non-radiographic axial spondyloarthritis, a protocol amendment was introduced in 2014 to include such patients into the registry. However, since only 9 GLM-treated patients (7.6%) did not have erosions on sacroiliac joints by X-ray, these patients were included in the full analysis set. No specific analyses were otherwise done on these nine GLM-treated patients with non-radiographic axial spondyloarthritis. Therefore, the term AS will be used throughout this manuscript. All analyses included the full analysis set comprising patients receiving treatment without major eligibility violations.

### Data collection

The following clinical, laboratory and patient-reported outcomes (PROs) were collected as per routine care: AS Disease Activity Score (ASDAS), BASDAI, BASFI [31], health assessment questionnaire (HAQ), patient global assessment (PtGA), physician global assessment (MDGA), back pain scores, enthesitis (as assessed by examining supraspinatus, medial epicondyle humerus, lateral epicondyle humerus, greater trochanter, quadriceps-to-patella, patellar-tibia, Achilles and Plantar Fascia sites), dactylitis (scored as present or absent), and acute phase reactants (CRP, ESR). Safety was assessed with the incidence of treatment-emergent adverse events (AEs).

### Statistical analysis

The current study includes data from two distinct statistical analysis plans. The first analysis plan covered the IFX cohort and was filed in May 2018 while the second covered the remainder of cohort and included patients treated with GLM. Since comparison of the two treatments was not within the scope of the registry and the investigators had already been exposed to the IFX data, a decision was made not to do any direct statistical analyses comparing the IFX and GLM cohorts. Rather, data from the two cohorts are simply contrasted as it provides an interesting vision of how each drug was used.

All outcomes (presented as observed) were assessed descriptively using the median and/or mean and standard deviation (SD), 95% confidence intervals (CI) of the mean for continuous variables, and frequency distributions for categorical variables. In order to assess potential differences over calendar time in the baseline profile of AS patients selected in routine care for treatment with IFX and GLM, variations in patient demographics and baseline characteristics across enrolment periods (2005–2008, 2009–2012, 2013–2015 and 2016–2017) were assessed using the Kruskal-Wallis test for continuous variables and the Chi-square for categorical variables. There was no imputation for missing data.

Kaplan-Meier (KM) survival analysis was used to assess the time to IFX and GLM discontinuation. AEs were coded using the Medical Dictionary for Regulatory Activities (MedDRA version 20.0), and the proportion of patients who experienced an AE along with incidence rates were summarized by preferred term (PT). Statistical analyses were conducted with SPSS 24.0 (SPSS Inc., Chicago, IL) and SAS 9.4 (SAS Institute, Cary, NC, USA).

## Results

Patient demographics and baseline characteristics are presented in Table 1. Among the 389 IFX- and 421 GLM-treated patients, proportion of males were 62.7 and 59.1%, mean age was 45.6 and 45.7 years and mean disease duration was 8.6 and 6.0 years, respectively. Most patients were bio-naive (> 82%).

**Table 1:** Patient Demographics and Baseline Characteristics

	IFX	GLM
**Number of Patients**	**389**	**421**
**Male Gender, n (%)**	244 (62.7%)	249 (59.1%)
**Mean (SD) Age, years**	45.6 (11.9)	45.7 (13.3)
**Mean (SD) Weight, Kg**	79.8 (18.4)	81.9 (18.3)
**Disease Duration**		
Mean (SD)	8.6 (9.8)	6.0 (10.1)
Median	4.0	1.6
**HLA B27, n/N (%)**	27/42 (64.2%)	47/62 (75.8%)
**Uveitis, n/N (%)**		
History	19/67 (28.3%)	53/369 (14.3%)
If yes, present?	3/19 (15.7%)	4/46 (8.7%)
**Psoriasis, n (%)**		
History	8/66 (12.1%)	58/376 (15.4%)
If yes, present?	6/8 (75.0%)	40/56 (71.4%)
**Inflammatory Bowel Disease, n (%)**		
History	11/67 (16.4%)	33/373 (8.8%)
If yes, present?	8/9 (88.9%)	21/32 (65.6%)
**Peripheral Arthritis, n (%)**		
History	20/67 (30.0%)	143/373 (38.3%)
If yes, present?	13/18 (72.2%)	115/137 (83.9%)
**Presence of Enthesitis (n/N, %)**	21/360 (5.5%)	135/340 (39.7%)
**Presence of dactylitis (n/N, %)**	8/241 (3.3%)	31/235 (13.2%)
**Previous Therapies (n, %)**		
NSAIDs	302, 77.6%	343, 81.5%
Corticosteroids	97, 24.9%	97, 23.0%
DMARDs	99, 25.4%	61, 22.8%
**Concomitant Therapies (n, %)**		
NSAIDs	251, 64.5%	281, 66.8%
Corticosteroids	44, 11.3%	51/421, 12.1%
MTX	79, 20.3%	41/421 (9.7%)
**Bio-naive, %**	91.3%	82.7%
**BASDAI**		
Available n	374	405
Median	6.5	6.5
Mean (SD, 95% C.I)	6.3 (2.2, 6.1–6.5)	6.1 (2.1, 5.9–6.3)
**BASFI**		
Available n	374	403
Median	6.3	5.6
Mean (SD, 95% C.I)	5.9 (2.5, 5.7–6.2)	5.3 (2.4, 5.1–5.6)
**PtGA**		
Available n	96	256
Median	64	70
Mean (SD, 95% C.I)	59.4 (27.8, 53.8–65.1)	61.6 (24.6, 58.5–64.6)
**MDGA**		
Available n	385	414
Median	7.0	6.0
Mean (SD, 95% C.I)	6.3 (2.1, 6.1–6.5)	5.5 (2.1, 5.3–6.0)
**HAQ**		
Available n	376	399
Median	1.1	1.0
Mean (SD, 95% C.I)	1.2 (0.6, 1.1–1.2)	1.0 (0.6, 1.0–1.1)
**CRP (mg/ml)**		
Available n	312	319
Median	9.0	5.9
Mean (SD, 95% C.I)	18.0 (27.8, 14.9–21.1)	14.8 (30.9, 11.4–18.2)
**ESR (mm/hr)**		
Available n	333	308
Median	18.0	12.0
Mean (SD, 95% C.I)	23.5 (20.7, 21.3–25.8)	17.2 (15.5, 15.4–18.9)
**ASDAS**		
Mean (SD, 95% C.I))	3.7 (1.1, 3.6–3.8)	3.4 (1.0, 3.3–3.5)
Median	3.8	3.4
Inactive (% < 1.3)	2.4%	2.7%
Moderate (% 1.3–2.0)	3.9%	7.5%
High (% 2.1–3.5)	35.5%	48.1%
Very High (% > 3.5)	58.3%	38.3%

Patients treated with IFX received a mean (SD) dose of 4.78 (1.38) mg/kg, over a median (min-max) of 17 (1–93) infusions representing a total exposure of 1251 years. All GLM-treated patients started at the 50 mg dose monthly and received a median (min-max) of 14 (1–85) injections representing a total exposure of 675 years. Additionally, among GLM treated patients, two received at least one 100 mg dose, 4 patients (1%) received 50 mg injections at shorter than q28 days intervals, while 50 patients (11.9%) received 50 mg injections at q28–32 days intervals throughout study.

**Fig. 1:**
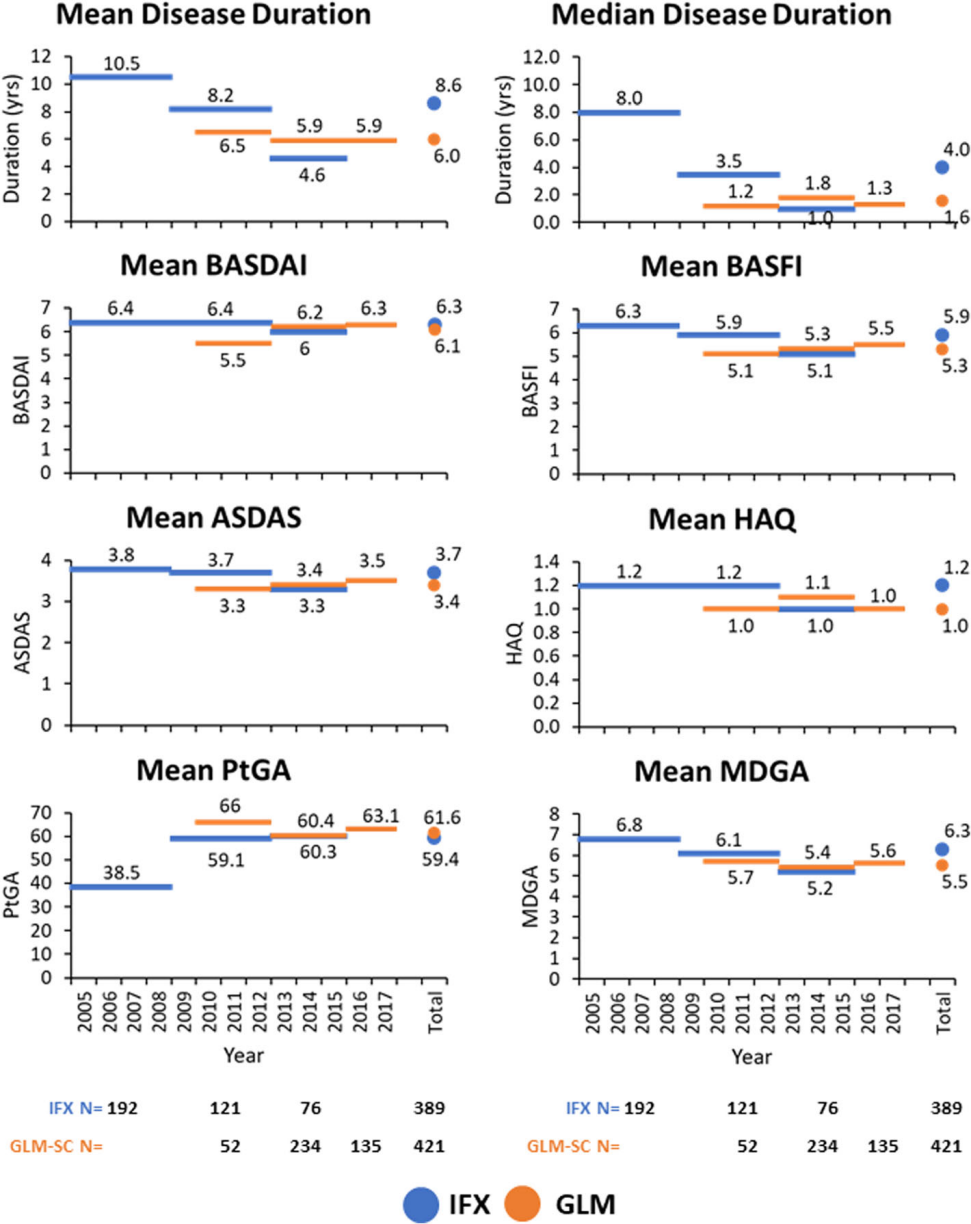
Evolution of baseline characteristics over time

As shown in Fig. 1, a significant decrease in baseline disease duration was observed in the IFX cohort from a median of 8.0 to 3.5 and 1.0 years in 2005–2008, 2009–2012 and 2013–2015, respectively (*p* < 0.001). A reduction in baseline mean BASFI score (6.3 vs. 5.9 vs 5.1; *p* = 0.011), MDGA score (6.8 vs 6.1 vs 6.2, *p* < 0.001), morning stiffness (78.4 vs 66.7 vs 46.9 min, p < 0.001), ESR (26.9 vs 20.2 vs 19.6 mm/hr., *p* < 0.003), CRP (18.8 vs 19.0 vs 13.8 mg/L, *p* = 0.045) and the proportion of patients in ASDAS very high disease activity (48.4, 43.8, 30.3%; *p* = 0.004) was also observed over the same time periods. As for GLM-treated patients, most disease parameters including median disease duration (1.6 years), mean baseline BASFI (5.3) and the proportion of patients in ASDAS very high disease activity (48%) remained similar from 2010 to 2017.

**Fig. 2:**
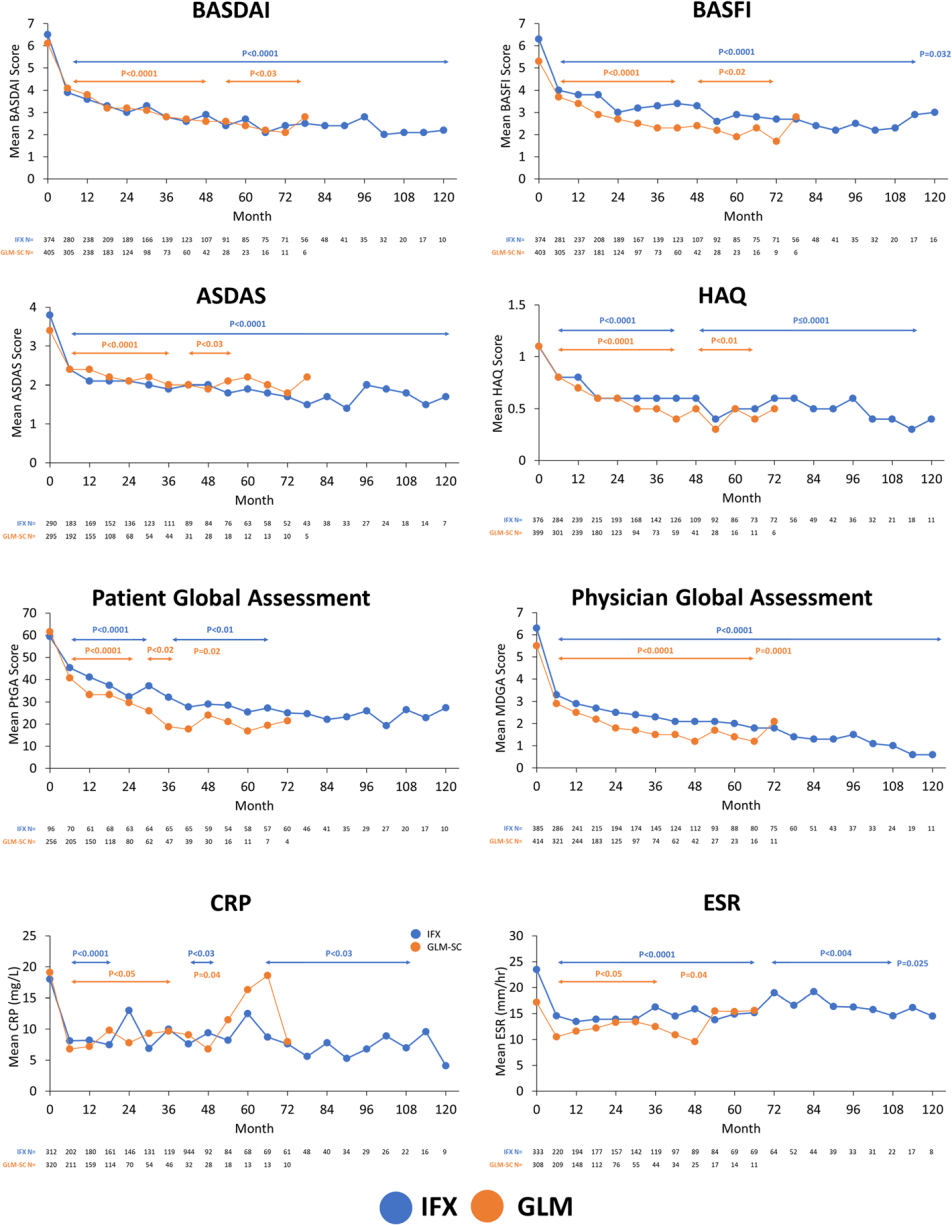
Effect of treatment with IFX and GLM on disease parameters over time. *P*-value vs baseline

**Fig. 3:**
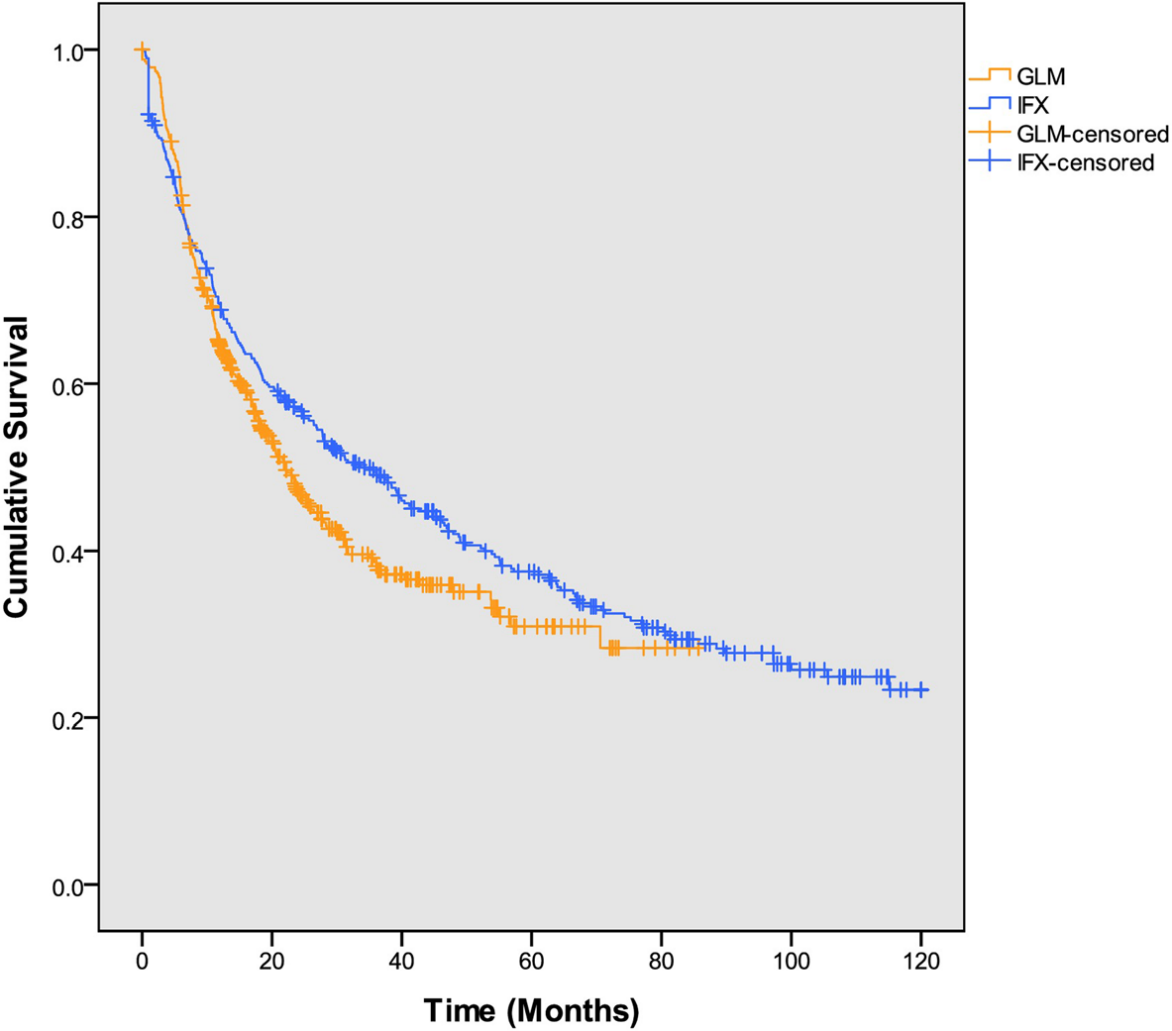
Kaplan-Meier drug survival analysis

Treatment with both IFX and GLM significantly improved BASDAI, BASFI, ASDAS, HAQ, CRP and ESR scores over time (*p* < 0.001) from baseline up to 120 and 84 months, respectively, with similar efficacy between agents (Fig. 2). The proportion of GLM-treated patients with enthesitis decreased from 135/340 (39.7%) to 51/234 (21.8%; *p* = 0.002) at 12 months and 16/124 (12.9%; *p* < 0.001) at 24 months. Similarly, the mean (SD) SPARCC enthesitis score decreased from 1.6 (2.93) at baseline to 0.6 (1.54) at 12 months (*p* < 0.001) and 0.3 (1.46) at 24 months (*p* < 0.001). The proportion of GLM-treated patients with dactylitis decreased from 31/235 (13.2%) to 6/203 (3.0%; *p* = 0.0209) at 12 months and 1/114 (0.9%; *p* = 0.005) at 24 months. The proportion of patients who discontinued treatment was 65.8% over a mean 3.2 years of exposure in the IFX cohort and 56.8% over 1.6 years in the GLM cohort (Fig. 3). The median estimated time to discontinuation was 33.6 and 22.1 months for IFX and GLM, respectively. In IFX-treated patients, the most common reasons for discontinuations were other (24.6%), adverse events (19.1%), lost to follow-up (14.1%), loss of response (14.1%) and lack of response (8.2%). For GLM-treated patients, the most common reasons for discontinuation were lack of response (33.9%), loss of response (18.8%), other (12.6%), lost to follow-up (11.7%) and adverse event (7.1%).

AEs were reported for 67.9 and 70.5% (136 and 131 events/100 PYs) and SAEs for 15.4 and 8.1% (10.5 and 8.45 events/100 PYs) covering 1251 and 675 years of exposure for IFX- and GLM-treated patients, respectively (Tables 2 and 3). The most frequently occurring AEs (> 7% of patient in either group) were drug ineffective, nasopharyngitis, upper respiratory tract infections, arthralgia and back pain. Discontinuation due to a SAE occurred in 17 (4.4%) and 7 (1.7%) IFX- and GLM-treated patients, respectively. The most commonly occurring SAEs in IFX-treated patients were osteoarthritis and therapeutic response decreased. The most commonly occurring SAE in GLM-treated patients was drug ineffective which occurred in 4 patients. The most common serious infection was pneumonia which occurred in two IFX-treated patients. There were 3 cases of opportunistic infections (three patients with candidiasis and one with latent TB) in IFX-treated patients, while one was observed in GLM-treated patients (onychomycosis). The incidence rate of malignancies was 0.89 and 1.93 per 100 pt.yrs. in IFX- and GLM-treated patients, respectively. There were three pregnancies in IFX-treated patients and seven in six GLM-treated patients (with one spontaneous abortion in a GLM-treated patient).

**Table 2:** Adverse events (SOC with PT terms occurring in ≥2% of patients with at least one agent)

Exposure (Total, Mean pt.yrs)	IFX (***n*** = 389)				GLM (***n*** = 421)			
	1251, 3.2				675, 1.6			
SOC/PT	N of Events	N of Patients	% of Patients	Rate/100 Pt-Yrs	N of Events	N of Patients	% of Patients	Rate/100 Pt-Yrs
**TOTAL**	**1687**	**264**	**67.9%**	**136**	**882**	**297**	**70.5%**	**131**
**Eye disorders**	**65**	**40**	**10.3%**	**5.24**	**28**	**22**	**5.2%**	**4.15**
Uveitis	15	9	2.3%	1.21	13	9	2.1%	1.93
**Gastrointestinal disorders**	**154**	**72**	**18.5%**	**12.4**	**56**	**39**	**9.3%**	**8.3**
Diarrhea	24	16	4.1%	1.93	13	12	2.9%	1.93
Dyspepsia	10	9	2.3%	0.81	3	3	0.7%	0.44
Nausea	27	19	4.9%	2.18	10	7	1.7%	1.48
Vomiting	13	12	3.1%	1.05	4	4	1.0%	0.59
**General disorders and administration site conditions**	**154**	**86**	**22.1%**	**12.4**	**176**	**156**	**37.1%**	**26.1**
Chest pain	18	13	3.3%	1.45	2	2	0.5%	0.3
Chills	17	8	2.1%	1.37	1	1	0.2%	0.15
Drug effect decreased	8	7	1.8%	0.64	27	26	6.2%	4.0
Drug ineffective	12	12	3.1%	0.97	90	88	20.9%	13.3
Fatigue	26	16	4.1%	2.10	10	9	2.1%	1.48
Pain	21	16	4.1%	1.69	0	0	0	0
Pyrexia	12	10	2.6%	0.97	6	5	1.2%	0.89
Therapeutic response decreased	8	8	2.1%	0.64	25	25	5.9%	3.7
**Infections and infestations**	**408**	**149**	**38.3%**	**32.9**	**309**	**129**	**30.6%**	**45.8**
Bronchitis	17	11	2.8%	1.37	16	16	3.8%	2.37
Ear infection	11	11	2.8%	0.89	13	13	3.1%	1.93
Gastroenteritis	17	15	3.9%	1.37	5	3	0.7%	0.74
Influenza	15	15	3.9%	1.21	10	8	1.9%	1.48
Nasopharyngitis	70	44	11.3%	5.64	59	31	7.4%	8.74
Pneumonia	22	18	4.6%	1.77	8	8	1.9%	1.19
Sinusitis	37	26	6.7%	2.98	27	18	4.3%	4.00
Tooth abscess	8	8	2.1%	0.64	3	3	0.7	0.44
Upper respiratory tract infection	36	28	7.2%	2.90	47	30	7.1%	1.33
Urinary tract infection	34	19	4.9%	2.74	9	9	2.1%	1.33
**Injury, poisoning and procedural complications**	**89**	**52**	**13.4%**	**7.17**	**29**	**25**	**5.9%**	**4.3**
Fall	12	10	2.6%	0.97	5	4	1.0%	0.74
Infusion-related reaction	30	16	4.1%	2.42	0	0	0	0
**Investigations**	53	38	9.8%	4.27	10	10	2.4%	1.48
Hepatic enzyme increased	12	11	2.8%	0.97	3	3	0.7%	0.44
**Musculoskeletal and connective tissue disorders**	**263**	**80**	**20.6%**	**21.2**	**65**	**45**	**10.7%**	**9.63**
Ankylosing spondylitis	11	10	2.6%	0.89	2	2	0.5%	0.30
Arthralgia	62	31	8.0%	5.00	9	7	1.7%	1.33
Back pain	53	28	7.2%	4.27	9	9	2.1%	1.33
Musculoskeletal pain	10	8	2.1%	0.81	2	2	0.5%	0.3
Neck pain	18	14	3.6%	1.45	3	3	0.7%	0.44
Pain in extremity	38	20	5.1%	3.06	8	8	1.9%	1.19
**Nervous system disorders**	**80**	**46**	**11.8%**	**6.45**	**34**	**29**	**6.9%**	**5.04**
Headache	27	21	5.4%	2.18	8	7	1.7%	1.19
Hypoaesthesia	22	12	3.1%	1.77	2	2	0.5%	0.3
**Respiratory, thoracic and mediastinal disorders**	**99**	**56**	**14.4%**	**7.98**	**27**	**19**	**4.5%**	**4.00**
Cough	20	17	4.4%	1.61	7	7	1.7%	1.04
Dyspnoea	12	11	2.8%	0.97	0	0	0	0
Oropharyngeal pain	22	12	3.1%	1.77	6	5	1.2%	0.89
**Skin and subcutaneous tissue disorders**	**119**	**63**	**16.2%**	**9.59**	**59**	**39**	**9.3%**	**8.74**
Pruritus	24	19	4.9%	1.93	0	0	0	0
Psoriasis	15	8	2.1%	1.21	12	9	2.1%	1.33
Rash	16	12	3.1%	1.29	9	9	2.1%	1.33
**Vascular disorders**	**50**	**30**	**7.7%**	**4.03**	**9**	**8**	**1.9%**	**1.33**
Hypertension	20	15	3.9%	1.61	6	6	1.4%	0.89

**Table 3:** Serious Adverse Events (SOC term) occurring in ≥0.5% of patients per agent

Exposure (Total, Mean pt.yrs)	IFX				GLM			
	1251, 3.2				675, 1.6			
SOC	N of Events	N of Patients	% of Patients	Rate/100 Pt-Yrs	N of Events	N of Patients	% of Patients	Rate/100 Pt-Yrs
**TOTAL**	**130**	**60**	**15.4%**	**10.5**	**57**	**34**	**8.1%**	**8.45**
Blood and lymphatic system disorders	0	0	0	0	1	1	0.2%	0.15
Cardiac disorders	13	8	2.1%	1.05	3	3	0.7%	0.44
Eye disorders	2	2	0.5%	0.16	1	1	0.2%	0.15
Gastrointestinal disorders	17	12	3.1%	1.37	7	6	1.4%	1.04
General disorders and administration site conditions	13	11	2.8%	1.05	6	6	1.4%	0.89
Hepatobiliary disorders	7	6	1.5%	0.56	0	0	0	0
Infections and infestations	16	12	3.1%	1.29	15	10	2.4%	2.22
Injury, poisoning and procedural complications	6	6	1.5%	0.48	4	3	0.7%	0.59
Metabolism and nutrition disorders	1	1	0.3%	0.08	2	2	0.5%	0.3
Musculoskeletal and connective tissue disorders	7	5	1.3%	0.56	2	2	0.5%	0.3
Neoplasms benign, malignant and unspecified (incl cysts and polyps)	6	6	1.5%	0.48	4	4	1.0%	0.59
Nervous system disorders	5	5	1.3%	0.40	2	2	0.5%	0.3
Psychiatric disorders	1	1	0.3%	0.08	2	2	0.5%	0.3
Renal and urinary disorders	4	3	0.8%	0.32	3	2	0.5%	0.44
Reproductive system and breast disorders	4	3	0.8%	0.32	1	1	0.2%	0.15

Two deaths occurred in IFX-treated patients (myocardial infarction; drowning) and two among GLM-treated patients (patient #1: oropharyngeal cancer; patient #2: Neutropenia, staphylococcal/pseudomonas infections, septic shock).

## Discussion

Although there are a substantial number of prospective registries evaluating the effect of anti-TNFs therapy in inflammatory arthritis, the majority of them follow only rheumatoid arthritis patients and collect predominantly safety and/or drug retention data [32]. Indeed, only a few multi-centre registries collect real-world prospective data on anti-TNF agents in AS patients and these include a number of national rheumatology registries from Scandinavian countries [33], GO-NICE from Germany [34, 35], ATTRA from the Czech Republic [36], the LORHEN registry in Northern Italy [37], the Korean registry OSKAR [38], the US-based CORRONA registry [39], BIOBADABRASIL [40] and, finally, the DEvenir des Spondylarthropathies Indifférenciées Récentes (DESIR) cohort in France [41]. Among them, BioTRAC is one of the oldest and longest-running AS drug registries. It has provided long-term effectiveness and safety data on patients treated with both older (IFX) and newer (GLM) anti-TNF agents, as well as insights on the evolution and treatment of the Canadian AS patient over the past two decades.

One advantage of long-term observational registries is that it permits the assessment of changes in treatment strategies over time, such as the reduction in baseline disease duration and disease activity between 2005 and 2009. This evolution in baseline characteristics likely results from changes in patient management involving the presence of enthesitis and dactylitis were respectively reported in 135/340 (39.7%) and 31/235 (13.2%) of GLM-treated patients, increased awareness of the disease and importance of earlier diagnosis and initiation of biologic therapy [26, 42]. Despite these improvements between 2005 and 2010, the BASDAI and ASDAS scores of Canadian AS patients from 2010 to 2016 remain high (range 3.5–6.3 and 2.7–3.8, respectively) [33] and the PtGA did not improve.

Among patients who were maintained on IFX and GLM, both anti-TNFs were equally effective in decreasing disease activity and improving function as the therapeutic response curves were superimposable despite differences in baseline disease activity indexes and retention. However, since the data presented is from “observed” patients, it could also be reflective of the disease state at which a therapy is deemed to be effective. In this and other registries, IFX-treated AS patients had longer treatment persistence compared to both GLM-treated AS patients and RA patients in general [37, 43]. The longer time to discontinuation observed in IFX-treated AS patients could be driven by low availability of alternative biologic therapies in earlier time periods since the reasons for discontinuation were more commonly “other”, “lost to follow-up” or “adverse event”. Indeed, most IFX-treated patients were enrolled between 2005 and 2010, while most GLM-treated patients were enrolled from 2013 to 2017 (Fig. 1).

The incidence of AEs and SAEs was also found to be similar between IFX and GLM, although there were some notable differences. Patients treated with IFX had a greater incidence of chest discomfort, chest pain, fatigue, headaches, pain, pyrexia, pain in extremities and pruritus compared to GLM-treated patients, all of which could be due to acute and delayed infusion reactions [44]. Conversely, GLM-treated patients had a greater incidence of “lack of response” or “loss of response” AEs compared to IFX-treated patients. Although this was likely driven by changes in the “End Of Participation” questionnaire, and the addition of lack/loss of response as an AE of special interest in a protocol amendment in 2014, which disproportionately impacted the GLM cohort as most patients were enrolled from 2013 onwards.

The incidence of serious infections for IFX- and GLM- treated AS patients was estimated between 1.29–2.22 events/100 pt.yrs., respectively. The only data available on the risk of serious infections in AS patients under anti-TNF therapy comes from randomized-controlled studies and meta-analyses, where the relative risk of serious infections was similar with controls [45]. The rates observed for AS patients in this report were similar to those found in RCTs [21, 23] and in rheumatoid arthritis and psoriatic arthritis patients in BioTRAC [29, 30]. The incidence of uveitis in IFX- and GLM-treated patients were similar to that reported previously in IFX-treated AS patient and lower than the 15.6/100 pt.yrs. incidence rate observed in placebo-treated patients [46].

The limitations of this registry are the absence of a control group treated with NSAIDs or non-biologic DMARDs, the inclusion of predominantly bio-naïve patients, the lack of radiographic data and the inherent biases and underreporting that are common within non-interventional, observational studies. Also, data completeness was quite variable over enrolment period due to protocol amendments, changes in standard operating procedures between the three study sponsors and improvements in adverse event reporting. Indeed, the IFX cohort had a substantial amount of missing data with respect HLA-B27 status, and assessment of extra-articular manifestations (including enthesitis and dactylitis), which limited the assessment of these variables.

One key strength of BioTRAC is that it included an extensive evaluation of clinical disease parameters and patient reported outcomes. Additionally, due to its long-term duration, BioTRAC offered a unique opportunity to evaluate the real-world effectiveness and safety of two anti-TNF agents in a community Canadian setting, while assessing regional variations due to differences in patient profiles, practice patterns and local reimbursement policies impacting access to care over 12 years. This makes the data more generalizable to the overall AS population than registration studies that tend to follow a more defined patient population using a rigid protocol over a shorter time period.

## Conclusions

In conclusion, differences in baseline characteristics over time suggest improvement in early diagnosis of AS and earlier access to biologic therapies. Both IFX and GLM treatment significantly reduced disease activity and improved functionality in a similar fashion, and were well tolerated in patients with AS.

## Data Availability

Janssen has an agreement with the Yale Open Data Access (YODA) Project to serve as the independent review panel for evaluation of requests for CSRs and participant level data from investigators and physicians for scientific research that will advance medical knowledge and public health. For more information on this process or to make a request, please go to https://yoda.yale.edu/.

## Abbreviations

AE: Adverse Event
AS: Ankylosing spondylitis
ASDAS: Ankylosing Spondylitis Disease Activity Score
AxSpA: Axial spondyloarthritis
BASDAI: Bath Ankylosing Spondylitis Disease Activity
BASFI: Bath Ankylosing Spondylitis Functional Index
bDMARD: Biologic DMARD
BioTRAC: Biologic Treatment Registry Across Canada
CI: Confidence interval
CRP: C-Reactive protein
DMARD: Disease-modifying antirheumatic drug
ESR: Erythrocyte sedimentation rate
GLM: Golimumab
IFX: Infliximab
HAQ-DI: Health Assessment Questionnaire Disease Index
MDGA: Physician global assessment of disease activity
PT: Preferred term
PtGA: Patient global assessment of disease activity
RCT: Randomized-controlled trial
SAE: Serious adverse event
SD: Standard deviation
SOC: System organ classes
SpA: Spondyloarthritis
TNFi: Tumor necrosis factor inhibitor
